# Turning up the heat on non-immunoreactive tumors: autophagy influences the immune microenvironment in pancreatic cancer

**DOI:** 10.1186/s12920-022-01371-0

**Published:** 2022-10-19

**Authors:** Si-Yuan Lu, Jie Hua, Jiang Liu, Miao-Yan Wei, Chen Liang, Qing-Cai Meng, Bo Zhang, Xian Jun Yu, Wei Wang, Jin Xu

**Affiliations:** 1 Department of Pancreatic Surgery, Fudan University Shanghai Cancer Center, No. 270 Dong’An Road, 200032 Shanghai, China; 2 Department of Oncology, Shanghai Medical College, Fudan University, Shanghai, China; 3 Shanghai Pancreatic Cancer Institute, No. 270 Dong’An Road, 200032 Shanghai, China; 4 Pancreatic Cancer Institute, Fudan University, Shanghai, China

**Keywords:** Pancreatic cancer, Autophagy, Bioinformatics analysis, Immune microenvironment

## Abstract

**Background:**

Autophagy regulators play important roles in the occurrence and development of a variety of tumors and are involved in immune regulation and drug resistance. However, the modulatory roles and prognostic value of autophagy regulators in pancreatic cancer have not been identified.

**Methods:**

Transcriptomic data and survival information from The Cancer Genome Atlas (TCGA) and Gene Expression Omnibus (GEO) databases were used to construct a risk score model. Important clinical features were analyzed to generate a nomogram. In addition, we used various algorithms, including ssGSEA, CIBERSORT, XCELL, EPIC, TIMER, and QUANTISEQ, to evaluate the roles of autophagy regulators in the pancreatic cancer immune microenvironment. Furthermore, the mutation landscape was compared between different risk groups.

**Results:**

Pan cancer analysis indicated that most of the autophagy regulators were upregulated in pancreatic cancer and were correlated with methylation and CNV level. MET, TSC1, and ITGA6 were identified as the prognostic autophagy regulators and used to construct a risk score model. Some critical clinical indicators, such as age, American Joint Committee on Cancer (AJCC) T stage, AJCC N stage, alcohol and sex, were combined with the risk model to establish the nomogram, which may offer clinical guidance. In addition, our study demonstrated that the low score groups exhibited high immune activity and high abundances of various immune cells, including T cells, B cells, and NK cells. Patients with high risk scores exhibited lower half inhibitory concentration (IC50) values for paclitaxel and had downregulated expression profiles of PD1, CTLA4, and LAG3. Mutation investigation indicated that the high risk groups exhibited a higher mutation burden and higher mutation number compared to the low risk groups. additionally, we verified our risk stratification method using cytology and histology data from our center, and the results are satisfactory.

**Conclusion:**

We speculated that autophagy regulators have large effects on the prognosis, immune landscape and drug sensitivity of pancreatic cancer. Our model, which combines critical autophagy regulators and clinical indicators, will provide guidance for clinical treatment.

**Supplementary Information:**

The online version contains supplementary material available at 10.1186/s12920-022-01371-0.

## Introduction

Pancreatic cancer remains one of the most malignant and lethal tumors, with 57,600 new cases of this disease and 47,050 deaths associated with pancreatic cancer reported in 2020 [1]. The lack of opportunity for early surgery and the scarcity of effective chemotherapy drugs pose challenges for pancreatic cancer therapy. Immunotherapy with immune checkpoint inhibitors (ICIs) has achieved ideal therapeutic results in various solid cancers [2]. However, ICIs exhibit negligible benefits in pancreatic cancer therapy due to the cold tumor immune microenvironment [2, 3]. In addition, many pancreatic cancer patients undergoing chemotherapy exhibit chemotherapeutic drug resistance to various degrees. Hence, developing a new method to detect the drug sensitivity of pancreatic cancer chemotherapy and immunotherapy is an urgent mission.

One of the difficulties that prevents therapeutic approaches from working effectively and causes the immune evasion of pancreatic malignant cells is the tumor microenvironment [4]. Due to the absence of a significant number of tumor-associated macrophages (TAMs), regulatory T cells (Tregs), myeloid-derived suppressor cells (MDSCs) [5–7], the microenvironment of pancreatic cancer is always immunosuppressive. Additionally, the biological effects of B cells and T cells are always inhibited by cancer cells, and normal immune functions are restrained [8].

Autophagy is a complex pathophysiological process that relies on autophagosomes and autolysosomes to recover cytoplasmic components or organelles [9]. The theory of autophagy was first proposed by Christian De Duve, and research interest in this field peaked in 2016, with the Nobel Prize for Medicine and Physiology awarded for autophagy-related research [10]. Autophagy has been found to play a significant role in the pathophysiological processes of many diseases, including diabetes [11], obesity [12], heart disease [13, 14], neurodegenerative disorders [15], and cancer [16]. Moreover, autophagy has dual functions in cancer: it can promote the survival of cancer cells or lead to their death [9]. In-depth study of the functions of autophagy regulators may reveal the underlying mechanisms of cancer metabolism and progression.

Recent studies have indicated that autophagy regulators participate in the modulation of immune activity and chemotherapy resistance [17, 18]. Pua et al. identified the critical role of autophagy in the growth, proliferation and differentiation of T cells [19]. Deficiencies in certain autophagy regulators, such as ATG7, can contribute to T cell mitochondrial metabolism disorders and cell cycle arrest [19, 20]. In addition, autophagy regulators can promote the differentiation of T cells into Tregs by regulating thymus differentiation [21, 22]. Cancer chemotherapy resistance also involves autophagy modulation. Some studies have shown that the induction of autophagy after chemotherapy can increase the sensitivity of patients to chemotherapy drugs and reduce drug resistance [18]. The death of autophagic cells may be the mechanism underlying this effect [23, 24]. Interestingly, most of these studies have indicated that the regulation of autophagy promotes tumor resistance through mechanisms that involve EGFR signaling, the PI3K/AKT/mTOR pathways [25], p53 [26], and MAPK14/p38a signaling [27]. However, despite the important role of autophagy regulators in pancreatic cancer therapy and prognosis, few studies have investigated their application prospects.

Gene mutations also play a critical role in the progression and evolution of pancreatic cancer, especially KRAS mutations and other key mutations in pancreatic cancer. We might find new directions by investigating the variables that influence these mutations [4].

Here, we performed a comprehensive bioinformatics analysis using The Cancer Genome Atlas (TCGA) and Gene Expression Omnibus (GEO) datasets to explore the expression profiles of autophagy regulators across cancers; the predictive values of risk model for the prognosis, immune landscape and the efficacies of therapeutic options (research design shown in Figure S1), with the goal of improving clinical therapeutic benefits.

## Materials and methods

### List of autophagy regulators and patient expression datasets

We identified 515 unique autophagy regulators in total, with 232 autophagy regulators from the Human Autophagy Database (HADb), 347 autophagy regulators from the Molecular Signatures Database (MSigDB), and 48 autophagy regulators from GeneCards. These autophagy regulators were used for subsequent analysis. In addition, we downloaded the gene expression data of 182 pancreatic cancer patients from TCGA-PAAD (RNA sequencing data, 182 samples) (https://cancergenome.nih.gov/) via the TCGAbiolinks R package. After vacancy value screening and clinical information integrating, we ultimately included 154 patients with complete clinical and expression information. For validation, GSE 62,452 (Microarray chip data, 66 samples) and PACA-AU (RNA sequencing data, 80 samples) was downloaded from GEO (https://www.ncbi.nlm.nih.gov/geo/) and used as a validation dataset.

### Autophagy regulator screening and determination of their expression levels in various tumors

To identify the most critical autophagy regulators in pancreatic cancer, we performed univariate Cox regression analysis, lasso regression and random forest analysis, and multivariate Cox regression analysis to progressively screen significant genes. Autophagy regulators that were correlated with patients’ overall survival (OS) were identified by univariate Cox regression analysis (R package “survminer 0.4.9”, R package “survival 3.3.7”), and *P* < 0.05 was considered significant. The lasso regression algorithm (R package “glmnet”, version 4.1.2, nfold = 1000, family = ‘cox’) can be used to reduce the coefficients of genes to 0 to facilitate variable selection. The random forest algorithm (R package “randomForestSRC” ,version 2.12.0, set.seed = 60, ntree = 100) takes the average or mode of the prediction results as the final data of a certain sample, which can reduce the prediction error. We applied the random forest and lasso regression algorithms as the second screening step. Then, multivariate Cox regression analysis (R package “survival 3.3.7”) was used to narrow down the numbers of selected genes and reduce the interactions among them. In addition, we analyzed the expression profiles of the selected genes in various tumors and investigated their effects on the prognosis of pancreatic cancer patients.

### Functional enrichment analysis of autophagy regulators

Gene Ontology (GO) and Kyoto Encyclopedia of Genes and Genomes (KEGG) [28] pathway analyses of the initially selected genes were performed with the R package org.Hs.eg.db 3.12 to explore the potential functions and pathways. The ggplot2 package of R was used to visualize the results.

### Construction of the prognostic risk score model and establishment of a nomogram

The genes remaining after the above screening process were used to construct a prognostic model. The risk scores were calculated and the patients were classified into high risk or low risk groups. Kaplan–Meier (K-M) curve, receiver operating characteristic (ROC) curve, and concordance index (C index) was utilized to evaluate the performance of the model.

### Immune activity and infiltration analysis

The stromal score, estimate score, immune score, and tumor purity of every sample were calculated using the estimate package. Various algorithms including CIBERSORT, QUANTISEQ, TIMER, ssGSEA, EPIC, and XCELL were applied to analyze the abundance of immune cells.

### Evaluation of the model effect for chemotherapy and immunotherapy

To further evaluate the significance of our model in pancreatic cancer, some analyses were performed to predict its efficacy in chemotherapy and immunotherapy. We calculated the half inhibitory concentration (IC50) scores of common chemotherapy drugs for pancreatic cancer (pRRophetic, version 0.5, batchCorrect = ‘eb’, minNumSamples = 10), and the Wilcoxon test was used to evaluate the differences between the high and low risk groups.

In addition, the relationship between immune checkpoints expression and model score was also explored, and violin plots and box plots were used to visualize the results.

### Mutation analysis

The R package “maftools” (version 2.6.05, rmOutlier = TRUE, addStat = ‘median’) was used to investigate the different mutation profiles of pancreatic cancer between different risk score groups.

### Cytological verification and histological verification

One human normal pancreatic epithelial cell line (H6C7) and four human pancreatic cancer cell lines (SW 1990, PANC-1, MIA-PaCa-2, CFPAC-1) were chosen to verify the expression levels of TSC1, ITGA6, and MET. All cell lines were cultured in Dulbecco’s modified Eagle’s medium (DMEM) with 10% fetal bovine serum (FBS) and 1% antifungal agent. We maintained each Petri dish in a humidified atmosphere at 37 °C with 5% CO2. Additionally, we identified the clinical value of the risk signature with data in Fudan University Shanghai Cancer Center (FUSCC). Total cDNA from 59 resected pancreatic cancer samples with good follow-up was extracted from the FUSCC cohort. The primer sequences for the above 3 genes and GAPDH were as follows: TSC1 forward: 5′- CAACAAGCAAATGTCGGGGAG-3′, TSC1 reverse 5′- CATAGGGCCACGGTCAGAA-3′; ITGA6 forward: 5′- ATGCACGCGGATCGAGTTT-3′, ITGA6 reverse: 5′- TTCCTGCTTCGTATTAACATGCT-3′; MET forward: 5′- AGCAATGGGGAGTGTAAAGAGG-3′, MET reverse: 5′- CCCAGTCTTGTACTCAGCAAC-3′; and GAPDH forward: 5′-CAGGAGGCATTGCTGATGAT-3′, GAPDH reverse: 5′-GAAGGCTGGGGCTCATTT-3′. We normalized our expression data by GAPDH and calculated the relative mRNA expression level by the 2^−ΔΔCt^ method.

## Results

### The genetic characteristics and transcriptional variations of key autophagy regulators in pan cancer and pancreatic cancer

**Fig. 1:**
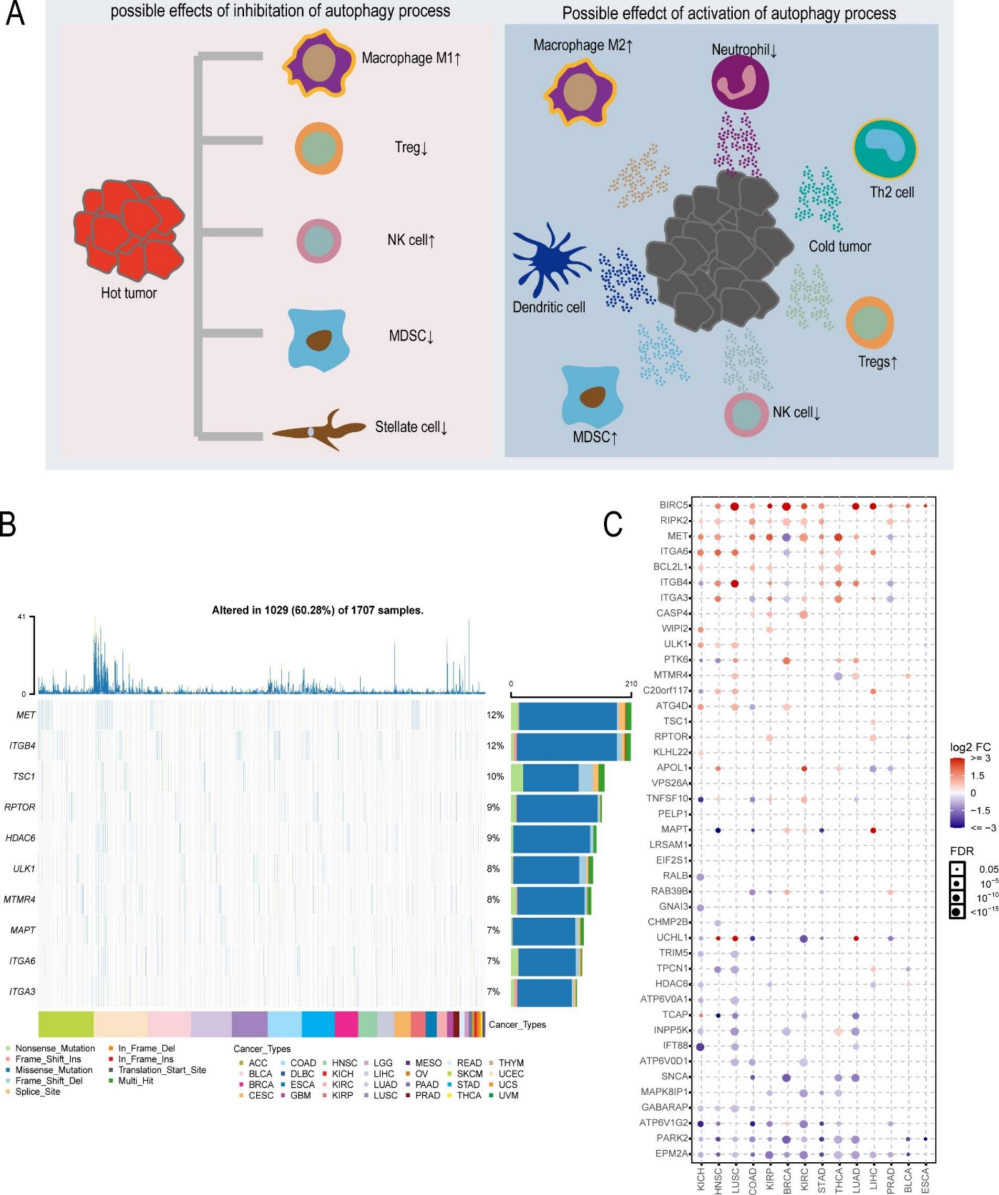
Expression variation of autophagy regulators. (**A**). The pattern map to show the potential role of autophagy in tumor immune microenvironment. (**B**) The waterfall plot shows the somatic mutations of the 10 autophagy regulators with the highest mutation rate using pan-cancer analysis. (**C**) Dot plot exhibits the expression level of these molecules between tumor and normal tissues.

We used multiple algorithms to identify the most influential autophagy regulators in our gene list. Forty-three autophagy regulators were identified by univariate Cox regression analysis (Figure S2). Figure 1 A exhibits the potential influence of autophagy in tumor immune microenvironment. We analyzed the mutational profiles of these autophagy regulators in the TCGA pan-cancer database, and found that 1707 patients had at least one mutation on these autophagy regulators. We selected the top ten molecules with highest mutation frequency and displayed them in waterfall plots (Fig. 1B). Patients with these molecules’ mutation accounted for 60.28% of the total samples, and the autophagy regulators with the highest mutation probability were MET (12%) and ITGB4 (12%). Methylation analysis indicated that the expression level of most autophagy regulators was negatively correlated with methylation level (Figure S3A). In addition, the CNV (Copy number variation) level of most autophagy regulators were positively correlated with gene expression (Figure S3B). We further compared the relative expression of these molecules in 14 cancer types containing paired tumor and normal samples and found that approximately half of the autophagy regulators showed elevated expression in different cancers, including BIRC5, RIPK2, MET, ITGA6, BCL2L1, CASP4, etc. (Fig. 1 C). We then screened 12 autophagy regulators by random forest and lasso regression to identify the most significant genes. Lasso regression confirmed 7 critical genes (TNFSF10, MET, CASP4, TPCN1, ATG4D, TSC1, BIRC5), and the random forest algorithm identified 8 essential genes (APOL1, ITGA6, MET, TNFSF10, VPS26A, EPM2A, RIPK2, CASP4). We combined the two results to obtain 12 significant autophagy regulators.

**Fig. 2:**
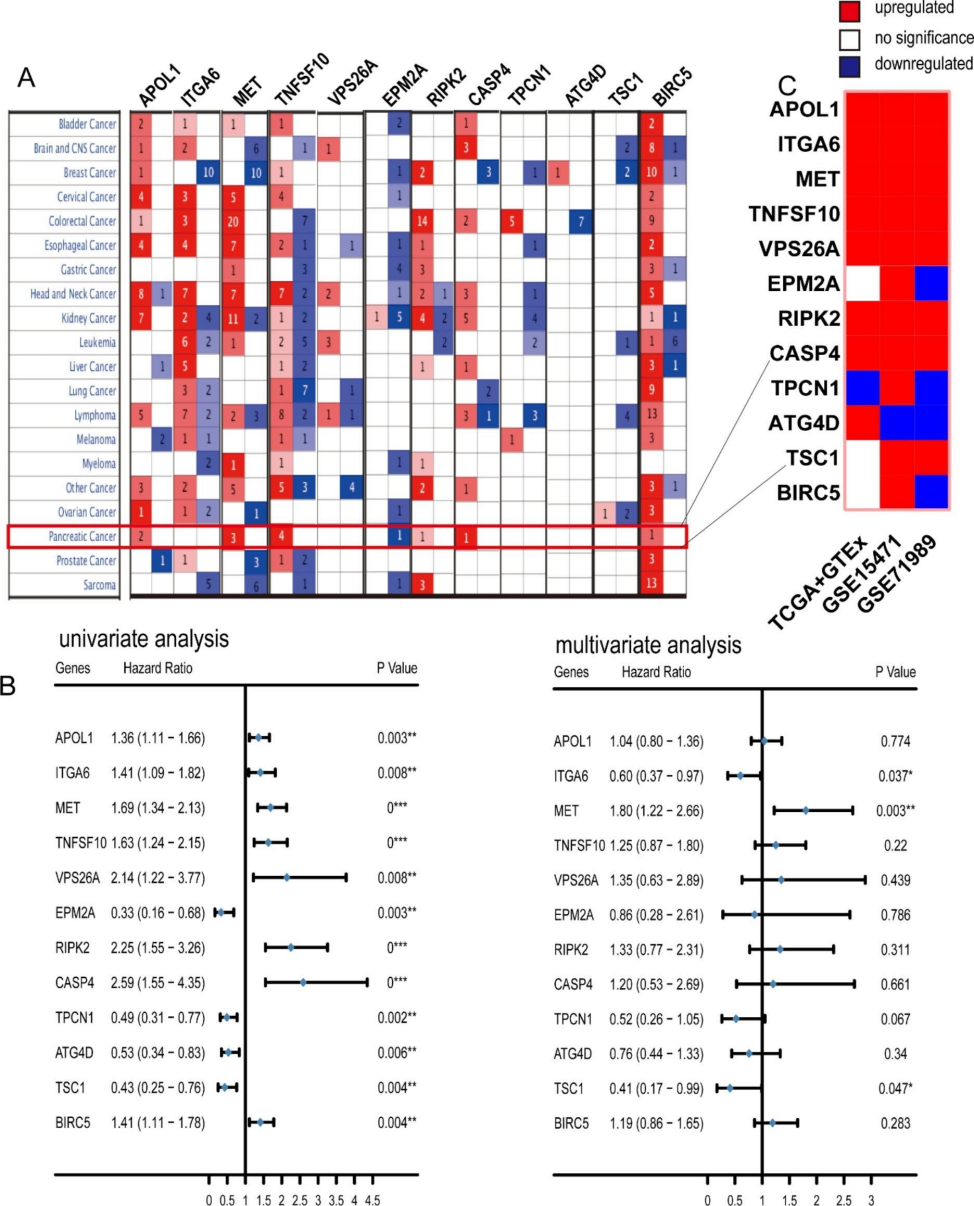
Expression profiles and survival significance of autophagy regulators. (**A**) Pancancer analysis of the expression profiles of 12 autophagy regulators. (**B**) Forest plots of univariate and multivariate analysis results showing the survival significance of 12 autophagy regulators. (**C**) The expression profiles of 12 autophagy regulators in various validated datasets of pancreatic cancer. The marked numbers in Fig. 2 A represent the number of datasets in which the gene was either up- or down-regulated in this cancer.

The pan cancer analysis suggested that these autophagy regulators also exhibited two expression trends in most cancers; most of these 12 autophagy regulators had upregulation trends in pancreatic cancer (Fig. 2 A). Additionally, these 12 autophagy regulators can significantly predict patient prognosis (Fig. 2B). Validation of these 12 identified autophagy regulators revealed a more notable effect. most of these 12 autophagy regulators were upregulated in pancreatic cancer, and 8 genes, including APOL1, ITGA6, MET, TNFSF10, VPS26A, RIPK2, CASP4, and TSC1, exhibited remarkably elevated expression (Fig. 2 C).

### Construction of a risk score model and nomogram

**Fig. 3:**
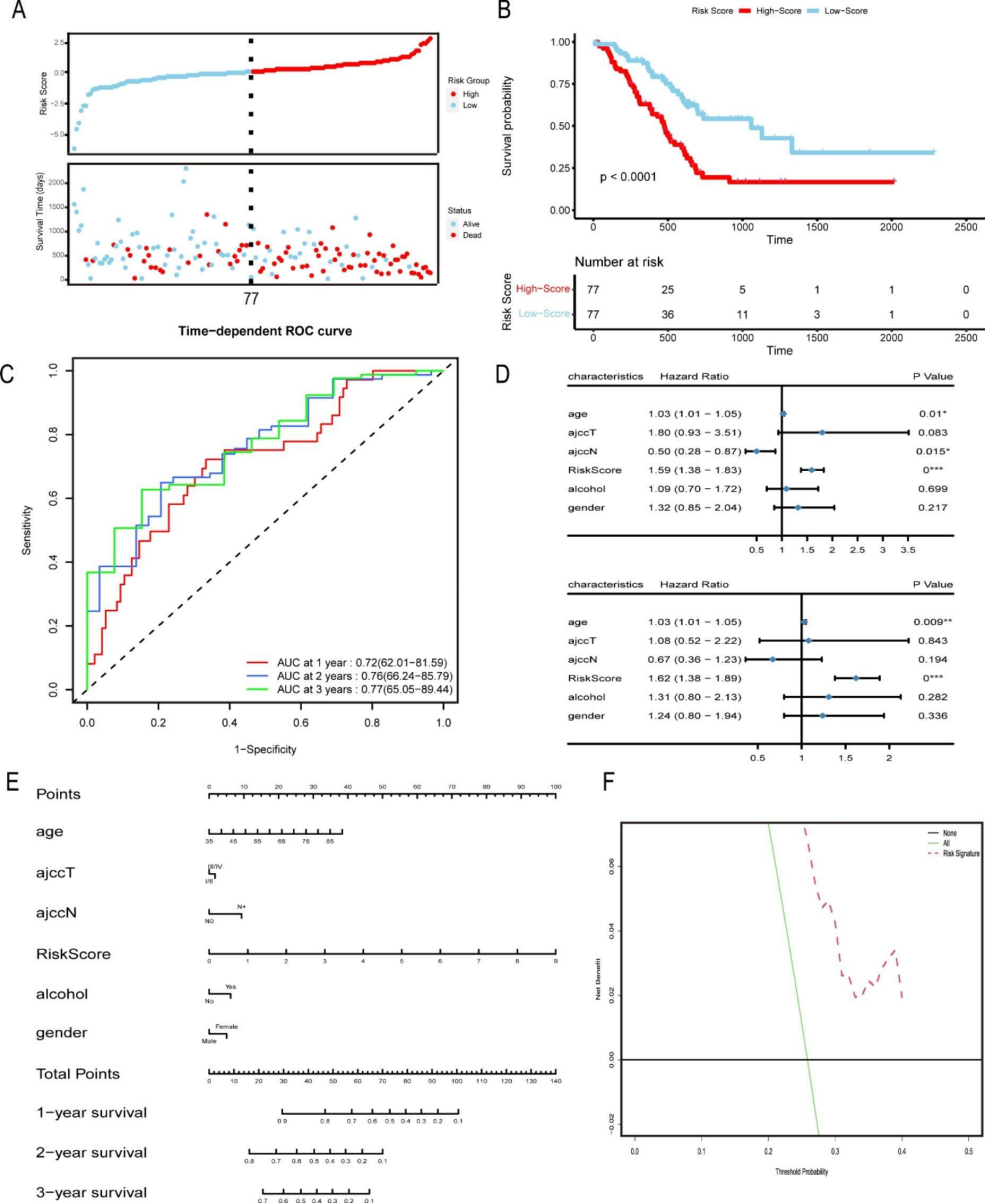
Construction of the autophagy-related risk score model and nomogram. (**A**) Risk score curve showing the distribution of each patient’s risk score and survival time. (**B**) The survival curve reflects the prognosis of patients with different risk scores. (**C**) The ROC curve verifies the accuracy of our risk score model. (D) Univariate and multivariate regression analyses of clinical characteristics and risk signatures. (**E**) Construction of a nomogram to accurately predict the prognosis of pancreatic cancer. (**F**) DCA curve showing the clinical benefit of our nomogram for pancreatic cancer patients.

To further investigate the predictive significance of autophagy regulators in pancreatic cancer, we constructed a risk score model based on TCGA. A total of 154 patients were selected after excluding patients with incomplete clinical information and removing NA values. Multivariate Cox regression analysis identified 3 autophagy regulators from the 12 identified genes that were strongly associated with pancreatic cancer prognosis: TSC1, ITGA6, and MET. The risk scores of all samples were calculated by the prediction algorithm utilizing the coefficient and expression data of each gene in the multivariate Cox regression model. All patients were divided into high risk and low risk groups according to their risk scores. The risk curve plot reflects each patient’s risk score and survival time (Fig. 3 A). The survival curve indicated that patients with low risk scores had elevated survival times and better prognoses than those with high risk scores (p < 0.001) (Fig. 3B). We then constructed a time-dependent ROC curve to validate the efficacy of our model, and the area under the curve (AUC) values at 1, 2, and 3 years were 0.72, 0.76, and 0.77, respectively (Fig. 3 C), indicating high predictive ability. A GEO dataset (GSE62452) was used as the validation cohort, and the AUCs at 1, 2, and 3 years were 0.55, 0.77 and 0.81, respectively, which suggested higher accuracy in the later 2 years (Figure S4A). As expected, the survival curve distinguished high risk and low risk samples, with a p value < 0.05 (Figure S4B). In addition, the prognostic role of the prediction model was also well validated in PACA-AU (Figure S4C-S4D). Considering the importance of clinical characteristics in predicting pancreatic cancer prognosis, we combined some clinical features with our risk score model to perform univariate and multivariate Cox regression analyses. The results indicated that the risk score were independent prognostic factors for pancreatic cancer (Fig. 3D). We then constructed a more comprehensive nomogram (Fig. 3E) using age, risk score, American Joint Committee on Cancer (AJCC) T stage, AJCC N stage, alcohol and sex. The DCA curve revealed the clinical benefits of our nomogram (Fig. 3 F).

### Investigation of the autophagy-associated mutation landscape in pancreatic cancer

**Fig. 4:**
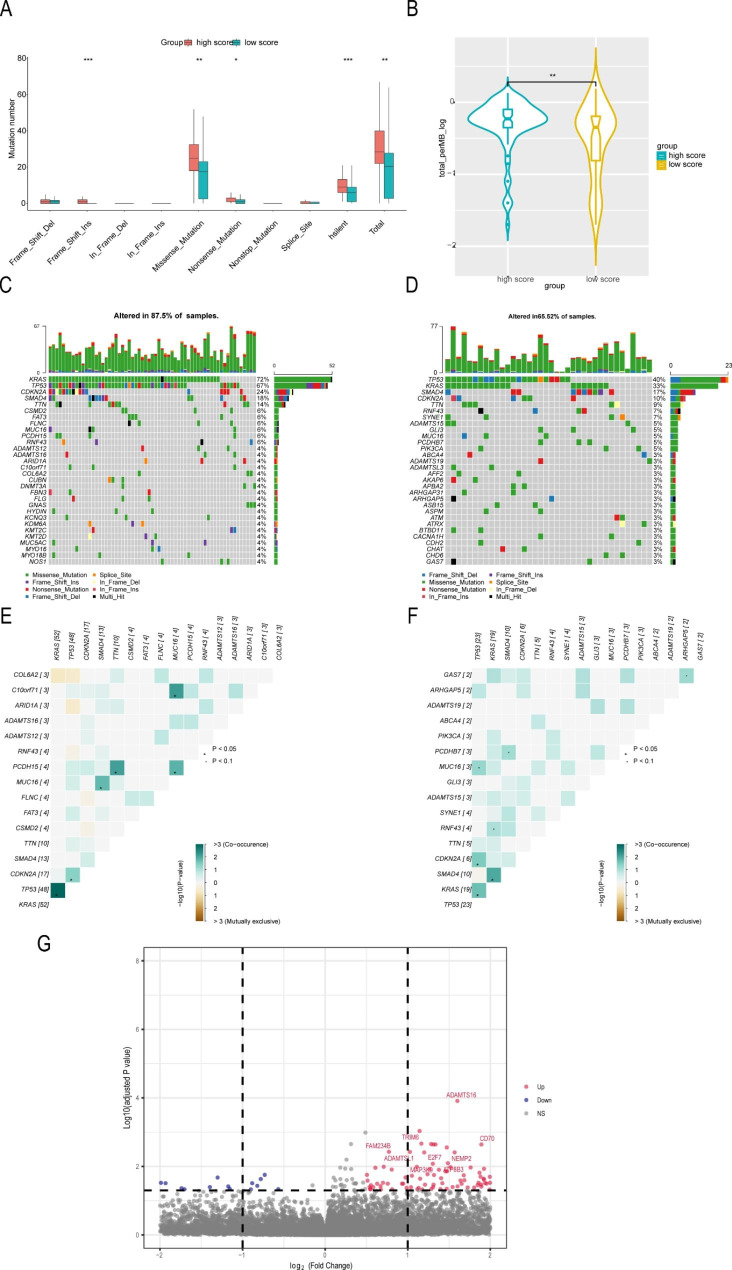
Investigation of the autophagy-associated mutation landscape in pancreatic cancer. (**A**, **B**) The high-risk group showed an obviously higher mutation burden and higher mutation numbers. (**C**) Oncoplot of the high-risk group. (**D**) Oncoplot of the low-risk group. (**E**) Comutation analysis of the high-risk group. (**F**) Comutation analysis of the low-risk group. (**G**) Differentially mutated gene analysis between samples with high and low scores.

We comprehensively compared the tumor mutation burden, mutation numbers, mutation panorama and co mutation status among different risk groups in TCGA. The high risk groups showed an obviously higher mutation burden and higher mutation numbers than the low risk groups (Fig. 4 A, 4B). The result was well validated in PACA-AU (Figure S4E). In addition, the high risk groups exhibited a higher mutation rate (87.5%) than the low risk groups (65.5%). Interestingly, the top five mutated genes were consistent among the different risk groups: TP53, KRAS, SMAD4, CDKN2A, and TTN (Fig. 4 C, 4D). Co mutation analysis identified some co mutation genes, including TP53 and CDKN2A in the high risk groups and SMAD4 and KRAS in the low risk groups (Fig. 4E F). In addition, we performed differentially mutated gene analysis between samples with high and low scores. We present the results of differentially mutated genes between the two groups using volcano plots, which are shown in Fig. 4G.

### Autophagy regulators play an important role in immune regulation

**Fig. 5:**
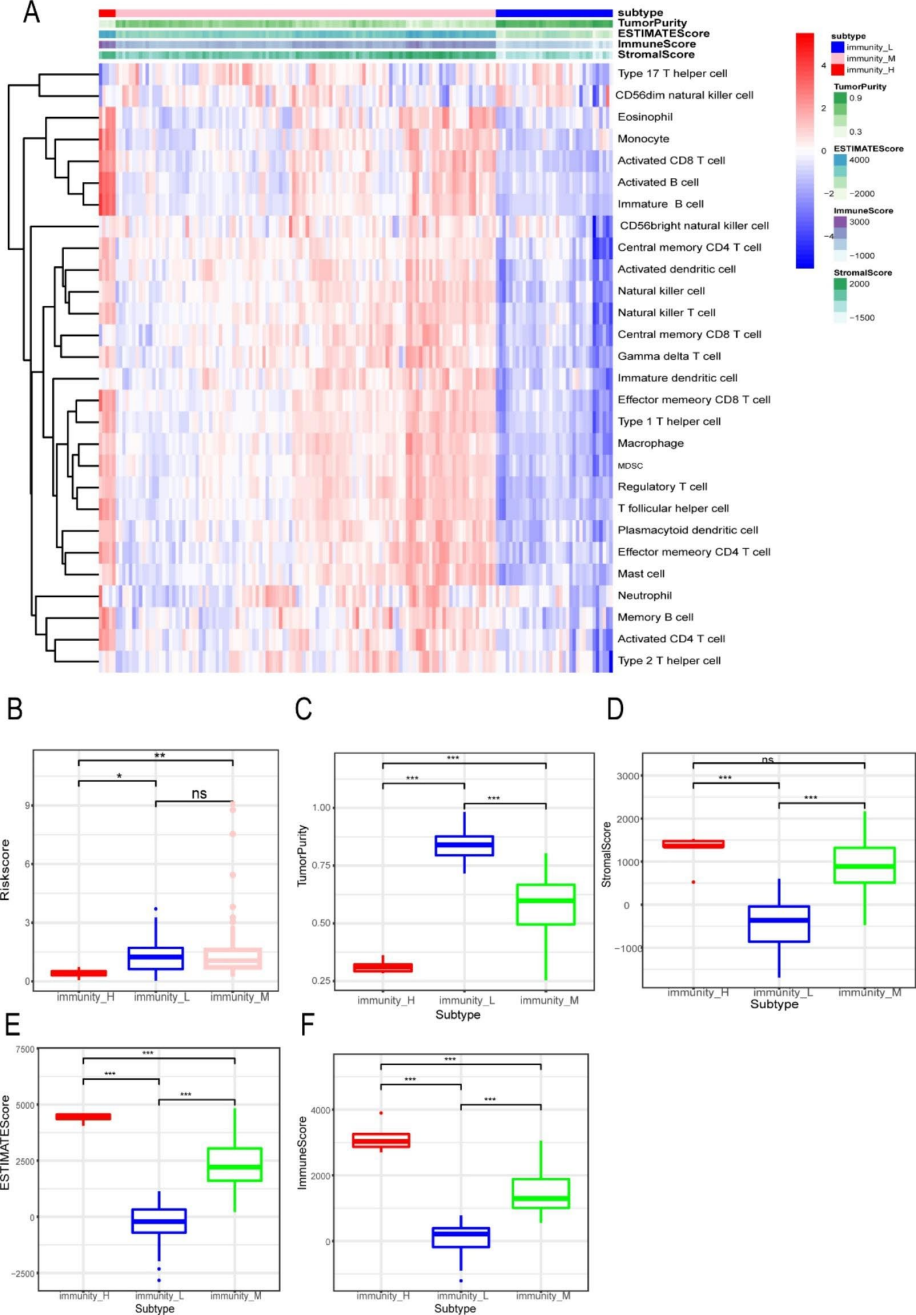
ssGSEA to perform immune activity stratification and the correlation with our risk model. (**A**) The estimation algorithm and ssGSEA were utilized to evaluate the immune activity of samples based on 28 immune cells. (**B**-**F**) The ability of our model to reflect the immune activity of the samples.

To investigate the relationships between autophagy regulators and immune cell activity in pancreatic cancer, we performed functional annotation of the expression matrix from TCGA-PAAD with 28 immune cell-related gene sets, and unsupervised clustering was used to demonstrate the results methodically. Patients were divided into 3 categories: low immune activity (immune-L), medium immune activity (immune-M), and high immune activity (immune-H) (Fig. 5 A). Then, we investigated the correlations between the autophagy regulators and immune activity. The immune-H group exhibited a lower risk score than the immune-M and immune-L groups (Fig. 5B). In addition, we calculated the stromal score, estimate score, immune score, and tumor purity of every sample and used these scores for analysis. The results indicated that immune activity was consistent with the stromal score, estimate score and immune score but contrary to tumor purity. Interestingly, different immune categories exhibited remarkable differences in stromal score, estimate score, immune score, and tumor purity (Fig. 5 C-5 F).

**Fig. 6:**
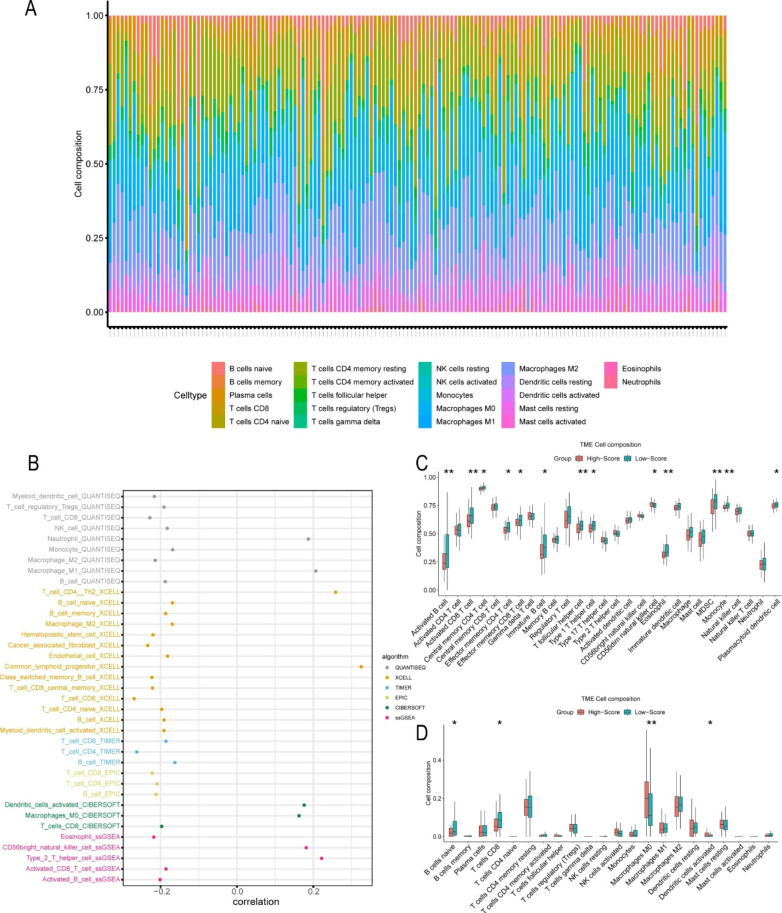
Immune cell infiltration and correlation analysis based on various algorithms. (**A**) A convolution histogram showing the proportions of immune cells in different samples (CICBERSORT). (**B**) A lollipop graph showing the correlations between immune cell scores and risk score. (**C**) Immune cell abundance differences between the high- and low-risk groups (ssGSEA) (**D**) Immune cell abundance differences between the high- and low-risk groups (CICBERSORT).

To explore the ability of our risk stratification method in assessing the activity of immune cells, algorithms including CIBERSORT, QUANTISEQ, TIMER, ssGSEA, EPIC, and XCELL were applied to analyze the abundance of immune cells (Fig. 6). A convolution histogram was constructed to show the proportions of immune cells in different samples (Fig. 6 A, CIBERSORT), and a lollipop graph was constructed to show the correlation between immune cell scores and risk scores (Fig. 6B, CIBERSORT, QUANTISEQ, TIMER, ssGSEA, EPIC, and XCELL). The results indicated that the abundances of T cells, B cells and NK cells estimated by different algorithms had strong correlations with our risk score (Fig. 6B). In addition, ssGSEA and CIBERSORT were performed to analyze the distribution of immune cells between the high and low risk groups. The low risk groups had more immune cell infiltration than that in the high risk group (Fig. 6 C, D). In addition, the prediction of immune infiltration by the predictive model was also well validated in PACA-AU (Figure S4F-S4G).

### Autophagy regulators have great potential for immune therapy and chemotherapy

**Fig. 7:**
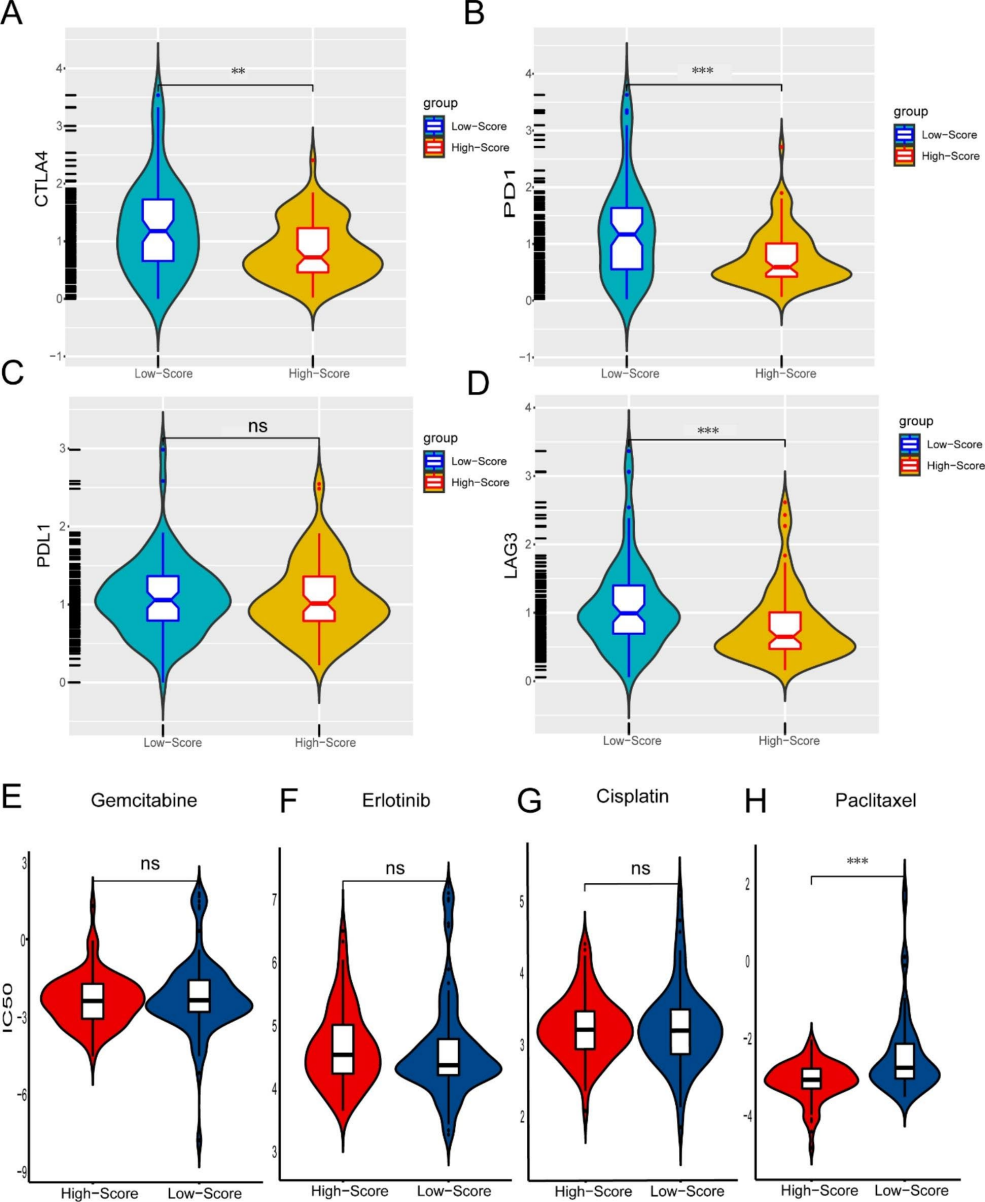
The potential predictive performance of the risk score model for chemotherapy and immune therapy. (**A**-**D**) Correlations between risk score and important immune checkpoint expression levels. (**E**-**H**) Ability of our risk score model to predict the IC50 of chemotherapy drugs.

We investigated the inner correlation between our risk score and therapy efficacy in pancreatic cancer to explore new prediction methods for prognosis (in TCGA database). We explored the relationship between ICI targets and our risk score model to identify the predictive potential. The results indicated that a low risk score was associated with relatively high expression levels of PD1 (p < 0.001), CTLA4 (p < 0.01), and LAG3 (p < 0.001), while PDL1 showed no significant association with risk score (Fig. 7 A-7D). we validated this result in PACA-AU and the conclusion was consist with that in TCGA (Figure S5A).

We the discovered that patients with lower risk scores exhibited a higher IC50 of paclitaxel (p < 0.001), while patients with lower risk scores exhibited a lower IC50 of other three drugs (Fig. 7E H, S5B), suggesting that our model is a potential indicator for drug sensitivity.

### Functional enrichment analysis

**Fig. 8:**
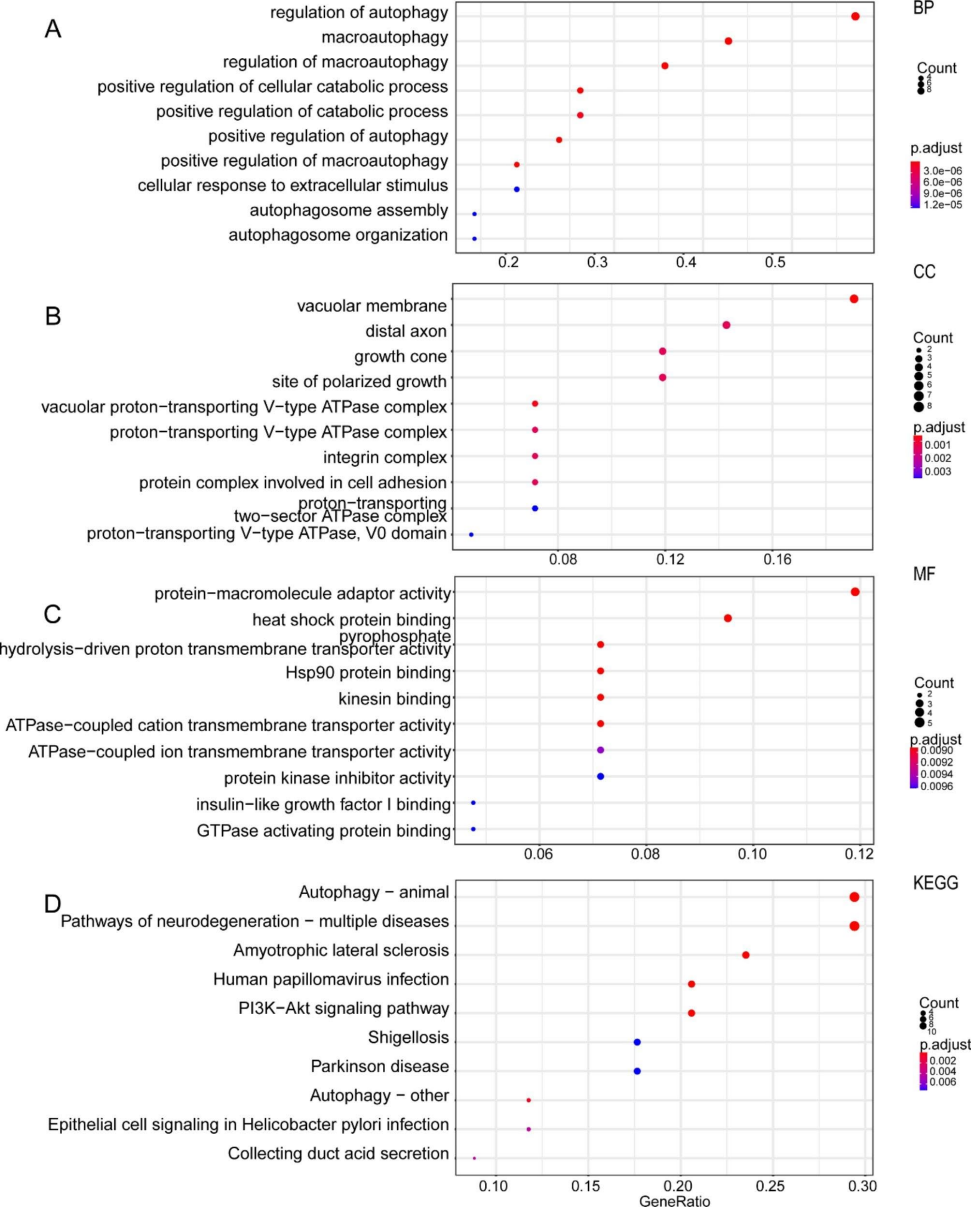
Functional enrichment analysis of autophagy regulators. (**A**) BP analysis of 43 autophagy regulators. (**B**) CC analysis of 43 autophagy regulators. (**C**) MF analysis of 43 autophagy regulators. (**D**) KEGG analysis of 43 autophagy regulators.

GO analysis (Fig. 8) indicated that the enriched terms of these autophagy regulators mainly included “regulation of autophagy”, “macroautophagy”, “positive regulation of cellular catabolic process” and “cellular response to extracellular stimulus”. KEGG analysis indicated that these autophagy regulators were involved in the autophagy-related pathway, pathways of infection by some bacteria and viruses, and some neurodegenerative disease-related pathways (Fig. 8). In addition, we compared samples with a high- vs. low-risk score and perform a GSEA analysis with the DEG results. The result indicated that these differential expression genes mainly are involved in “leukocyte chemotaxis”, “cellular response to cytokine stimulus” and other immune related pathways, as well as some tumor-related process including, “angiogenesis” and “positive regulation of cell motility” (Figure S6).

### Cytological verification and tissue sample verification

**Fig. 9:**
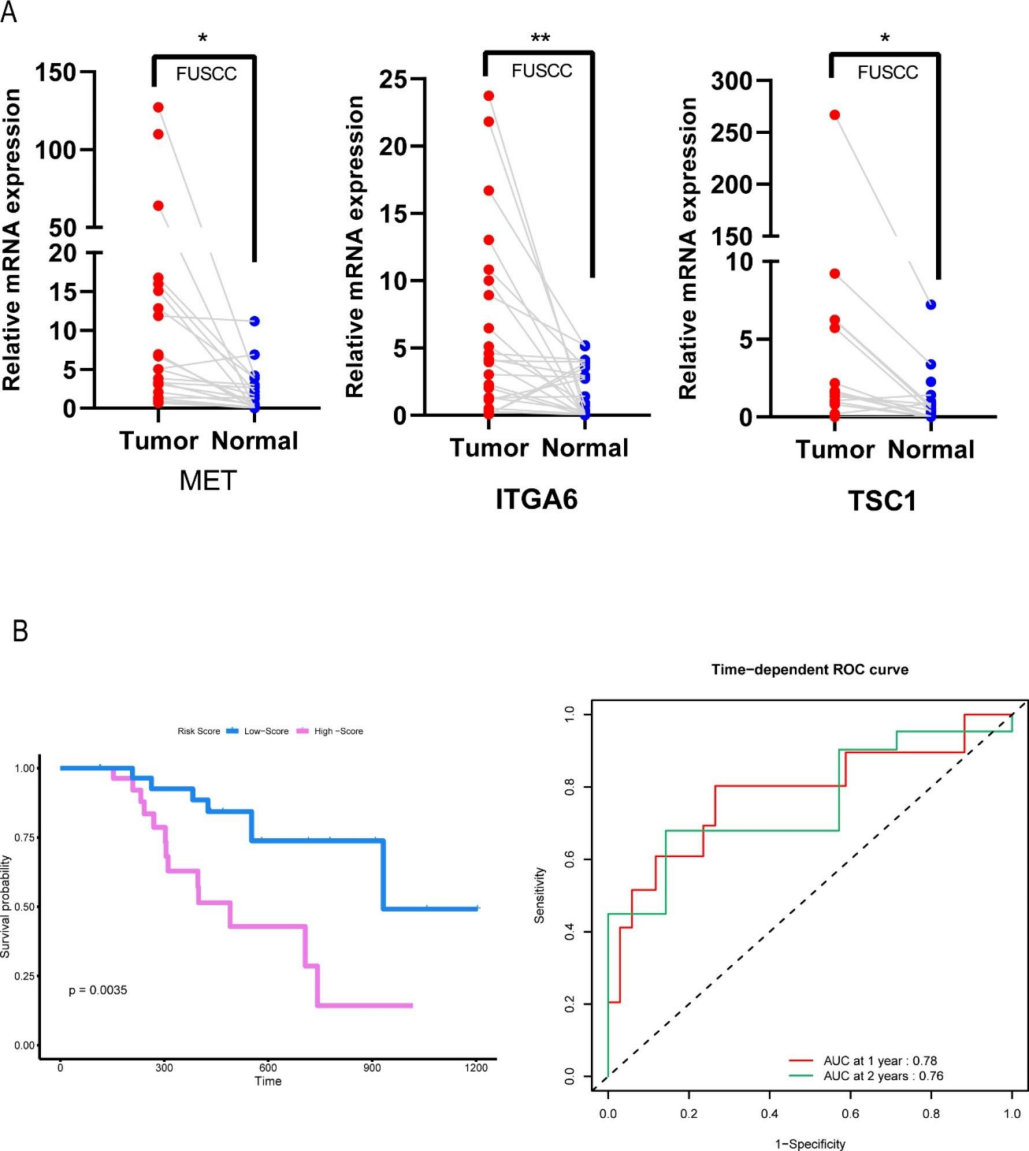
Verification of the clinical value of the risk signature in FUSCC. (**A**) The expression levels of TSC1, MET, and ITGA6 in cancer samples were higher than those in adjacent normal samples. (**B**) The survival time of high-risk patients was significantly lower than that of low-risk patients.

qRT-PCR was performed to detect the relative expression levels of TSC1, ITGA6, and MET in normal and cancer cell lines. The results (Figure S7) indicated that the expression levels of TSC1, ITGA6, and MET in most cancer cell lines (SW1990, PANC1, Mia-paca-2, CFPAC1) were significantly higher than those in the normal cell line (H6C7). In addition, according to the results from our 24 pairs of cancer and adjacent-tissue samples, the expression levels of TSC1, MET, and ITGA6 in cancer were significantly higher than those in adjacent normal tissues (Fig. 9 A). Our model was well validated in the FUSCC cohort. The survival time of high risk patients was significantly lower than that of low risk patients, with AUCs of 0.78 and 0.76 for one and two years, respectively (Fig. 9B).

### Immunohistochemistry verification

We obtained the protein expression profiles of key autophagy regulators from the Human Protein Atlas (HPA) database, which are shown in Figure S8. The immunohistochemical staining of tumor tissues was much more intense than that of normal tissues.

## Discussion

The development of bioinformatics tools has advanced oncology research. High-throughput genome sequencing and microarray technology can eliminate the limitations of single-gene studies and facilitate an understanding of the pathogenesis and prognosis of diseases at the level of the entire genome [29]. Many scientists have identified genetic signatures to evaluate tumor therapy and prognosis using bioinformatics methods [30]. In this study, we confirmed several significant autophagy regulators to construct a predictive model and identified their roles in influencing the prognosis, immune landscape and the efficacies of drug therapies. More importantly, our investigation established an accurate model by a novel screening method and is the first to evaluate the influence of autophagy regulators on the immune microenvironment of pancreatic cancer.

To build a model with high predictive ability and accuracy based on autophagy regulators, the choice of variables and screening process are the most important factors to consider. Cox analysis is a common method in bioinformatics that can incorporate survival time and outcome to screen variables while eliminating the interference of multicollinearity [31]. Zhang et al. identified tumor microenvironment (TME)-related genes in hepatocellular carcinoma using Cox analysis and built a risk model beneficial to patients [32]. In addition, Chen et al. utilized Cox analysis to validate the most important clinical indicators and obtained accurate results regarding outcomes [33]. In our study, we adopted a new screening method with multiple processes and algorithms to confirm the integrity of the prognostic model. Univariate Cox regression analysis was used to initially identify autophagy regulators that significantly influenced survival time and status, while multivariate Cox regression analysis was used to exclude the influences of gene interactions and collinearity. The novelty of our investigation method lies in the simultaneous use of lasso regression and the random forest algorithm to carry out the intermediate screening process and obtain the intersecting genes to ensure that critical regulators are not censored. The survival curve analysis indicated that high risk patients are highly differentiated from low-risk patients. In addition, the AUC values (> 0.7) at 3 years indicated that the prognostic model was accurate, and the DCA curve suggested a remarkable benefit to patients with pancreatic cancer. The prognostic model we constructed can predict precise outcomes of pancreatic cancer patients and may have broad application prospects in clinical practice.

Recently, TME investigation has become a focus of oncology research and may enhance our understanding of the crosstalk between cancer development and immune cells. In addition, by influencing immune cell infiltration, the TME may influence the therapeutic effects of immune therapy [34]. The tumor microenvironment is one of the challenges that hinders therapeutic methods from operating well and contributes to the immune evasion of pancreatic malignant cells [4]. However, autophagy regulators, as significant modulators of immune cell infiltration, have not been systematically evaluated in the pancreatic cancer immune microenvironment. Herein, we evaluated the correlation between autophagy related risk score and the abundances of immune infiltration cells to explore the roles of autophagy regulators. We found that our risk scores were significantly correlated with immune activity. The ssGSEA outcome indicated that a high-risk score was associated with a low degree of immune cell infiltration, indicating that our model has ideal immune evaluation ability. Based on the above results, we speculated that autophagy regulators were associated with the immune landscape of the pancreatic cancer microenvironment. However, the underlying molecular mechanism integrating autophagy and pancreatic cancer immunity remains to be elucidated. Some scientists have begun to explore the feasibility of utilizing autophagy modulators to enhance the immunotherapy of malignant carcinomas [35]. Choi et al. reported that SYK induced autophagy by promoting the production of reactive oxygen species (ROS) and the activity of MAPK8/9, which subsequently induced the surface expression of MHCII and CD4 + T cell activities, ultimately leading to remarkable increases in antitumor immunotherapy effects [36]. However, the application of autophagy in pancreatic cancer immunotherapy remains scarce. In our current study, we demonstrated a strong correlation between risk scores and different immune checkpoints, illustrating the feasibility of deciphering immunotherapy from an autophagy related perspective.

Chemotherapy is the primary therapeutic method for patients with pancreatic malignancies, either before or after surgery. Nevertheless, resistance to chemotherapeutic drugs often occurs in pancreatic cancer, leading to unsatisfactory treatment efficacy [37]. Here, we evaluated the correlations between chemotherapeutics, such as paclitaxel, gemcitabine, cisplatin and erlotinib, and the autophagy risk signature. High risk scores were associated with lower IC50 values for paclitaxel, while the other drugs showed no associations. Previous studies have proven the dual role of autophagy in inducing drug sensitivity. For example, Mirzoeva et al. demonstrated that chloroquine (an autophagy inhibitor) can promote the antitumor effects of PI3K/mTOR inhibitors in pancreatic cancer therapy [38]. In contrast, Torres et al. found that cannabinoids can remarkably activate autophagy-induced tumor cell death to enhance the antitumor activity of temozolomide [24]. Hence, we speculate that the mechanism underlying the high sensitivity to paclitaxel may involve the induction of autophagy regulators. Clinical investigations are imperative to test this hypothesis.

Our research has some limitations. First, the patient information used to build the model is from TCGA, while the validation set was derived from the GEO database. We did not validate our results comprehensively in samples from multiple centers. In addition, we have not verified our results in vivo and in vitro. Finally, the molecular mechanism by which autophagy regulators affect prognosis has not been determined, and further studies are needed to identify the important roles of autophagy regulators in pancreatic cancer at the molecular level.

In conclusion, our study fills the gap in predicting the prognosis of pancreatic cancer by autophagy regulators. The prognostic model we constructed has a strong predictive ability for the prognosis of pancreatic cancer patients and is correlated with the immune microenvironment in pancreatic cancer. We hope that these findings can provide some guidance for clinical prognosis prediction and for monitoring drug resistance.

## Electronic supplementary material

Below is the link to the electronic supplementary material.

Supplementary Material 1

Supplementary Material 2

## Data Availability

The access to all databases used in the present study were public available. GEO: (https://www.ncbi.nlm.nih.gov/geo/); TCGA: (https://portal.gdc.cancer.gov/);

## Abbreviations

ICIs: immune checkpoint inhibitors.
HADb: Human Autophagy Database.
MSigDB: Molecular Signatures Database.
OS: overall survival.
GO: Gene Ontology.
KEGG: Kyoto Encyclopedia of Genes and Genomes.
ROC: receiver operating characteristic.
C index: concordance index.
DCA: decision curve analysis.
ssGSEA: single-sample gene set enrichment analysis.
CNV: copy number variation.
BP: biological process.
CC: cellular component.
MF: molecular function.
