# Supplementary material for: Turning up the heat on non-immunoreactive tumors: autophagy influences the immune microenvironment in pancreatic cancer
Source: BMC Med Genomics. 2022 Oct 19;15:218. doi: 10.1186/s12920-022-01371-0 (PMC9580150; doi:10.1186/s12920-022-01371-0)
Supplement: Supplementary file 1 — Supplementary Material 1 [file 12920_2022_1371_MOESM1_ESM.docx]

**Figure S1** Research design and analysis process

**Figure S2** Forest plots of univariate analyses showing the survival significance of 43 autophagy regulators.

**Figure S3** Genetic variation of autophagy regulators. (A). The bubble chart exhibits the correlation between gene expression and methylation. (B). The bubble chart exhibits the correlation between gene expression and CNV.

**Figure S4** Validation of the risk score model in GSE62452 and PACA-AU. (A) ROC curve used to identify the accuracy of our model with the GSE62452. (B) Survival curve validated by our model with GSE62452. (C) ROC curve used to identify the accuracy of our model with PACA-AU. (D) Survival curve validated by our model with PACA-AU. (E) validation of the tumor mutation burden of different groups in PACA-AU. (F-G) validation of the immune infiltration in PACA-AU.

**Figure S5** validation of the predictive performance of the risk score model for chemotherapy and immune therapy in PACA-AU. (A) Correlations between risk score and important immune checkpoint expression levels. (B) Ability of our risk score model to predict the IC50 of chemotherapy drugs.

**Figure S6** GSEA analysis with the DEG results. (A) GSEA GO result of the DEG results. (B) GSEA KEGG result of the DEG results.

**Figure S7** qRT-PCR verification of the mRNA expression of TSC1, ITGA6, and MET.

**Figure S8** Immunohistochemistry verification of the protein expression of TSC1, ITGA6, and MET.
