# Supplementary figures and images for: Turning up the heat on non-immunoreactive tumors: autophagy influences the immune microenvironment in pancreatic cancer
Source: BMC Med Genomics. 2022 Oct 19;15:218. doi: 10.1186/s12920-022-01371-0 (PMC9580150; doi:10.1186/s12920-022-01371-0)

Figure s1


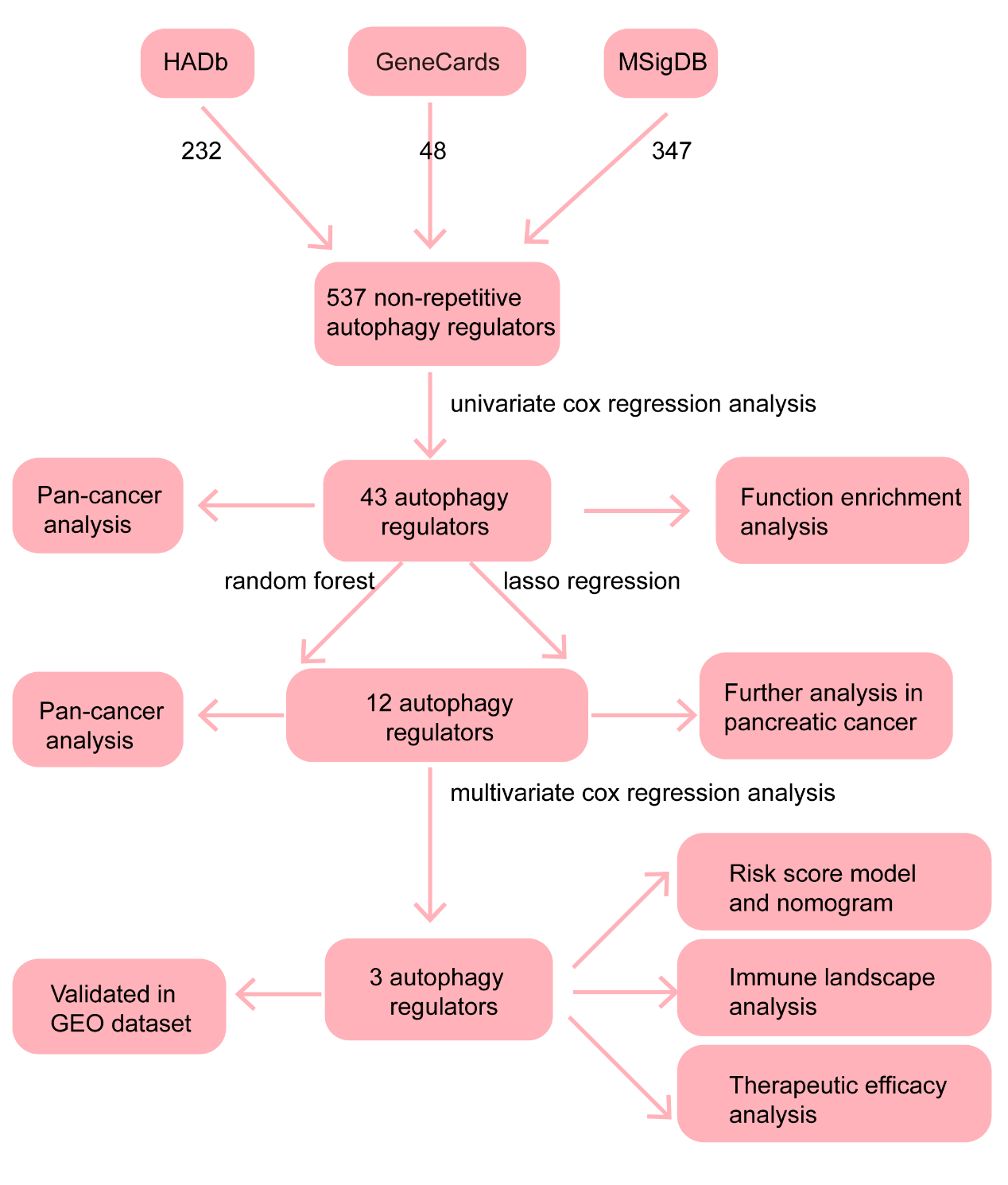


Figure s2


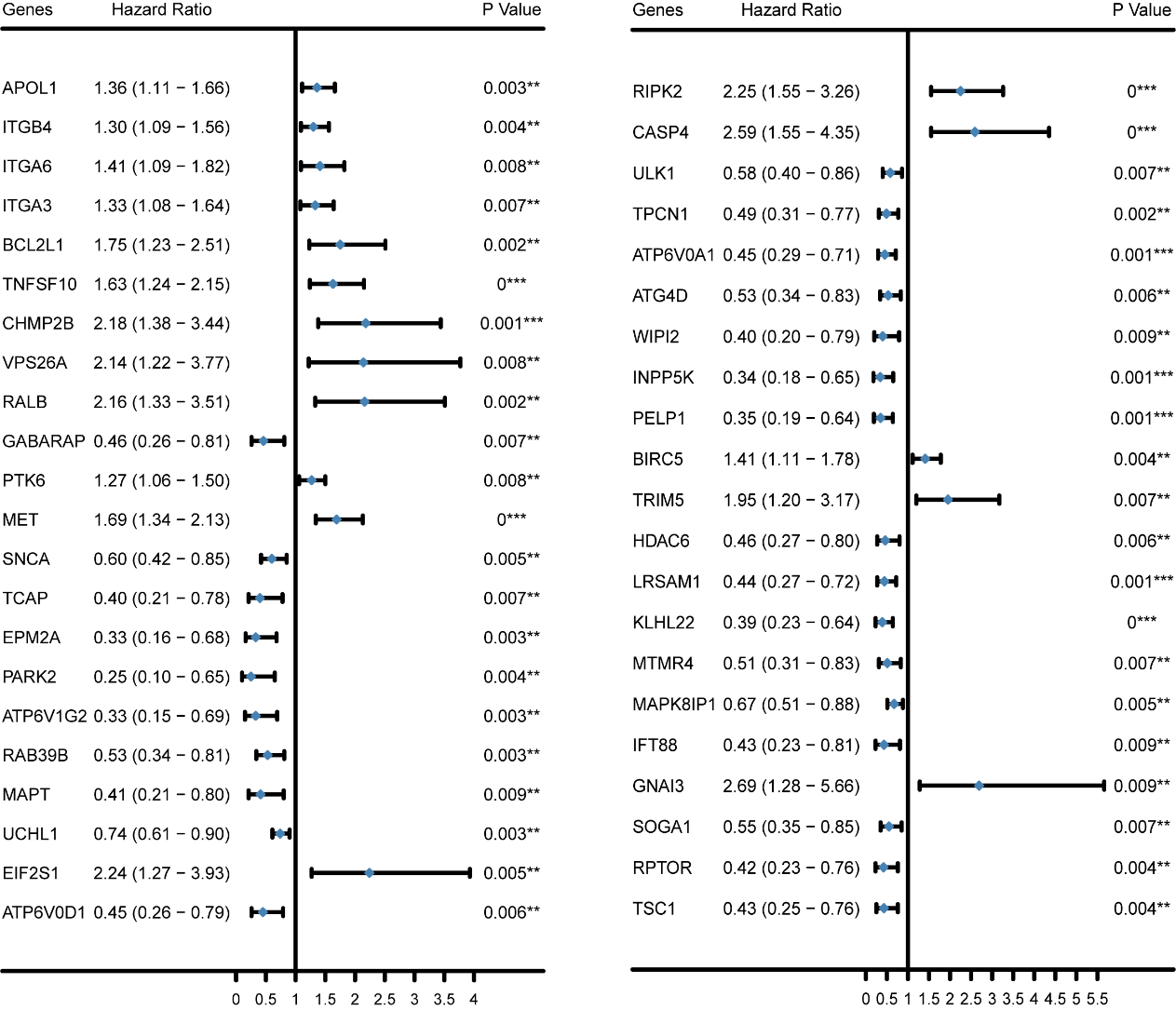


Figure s3


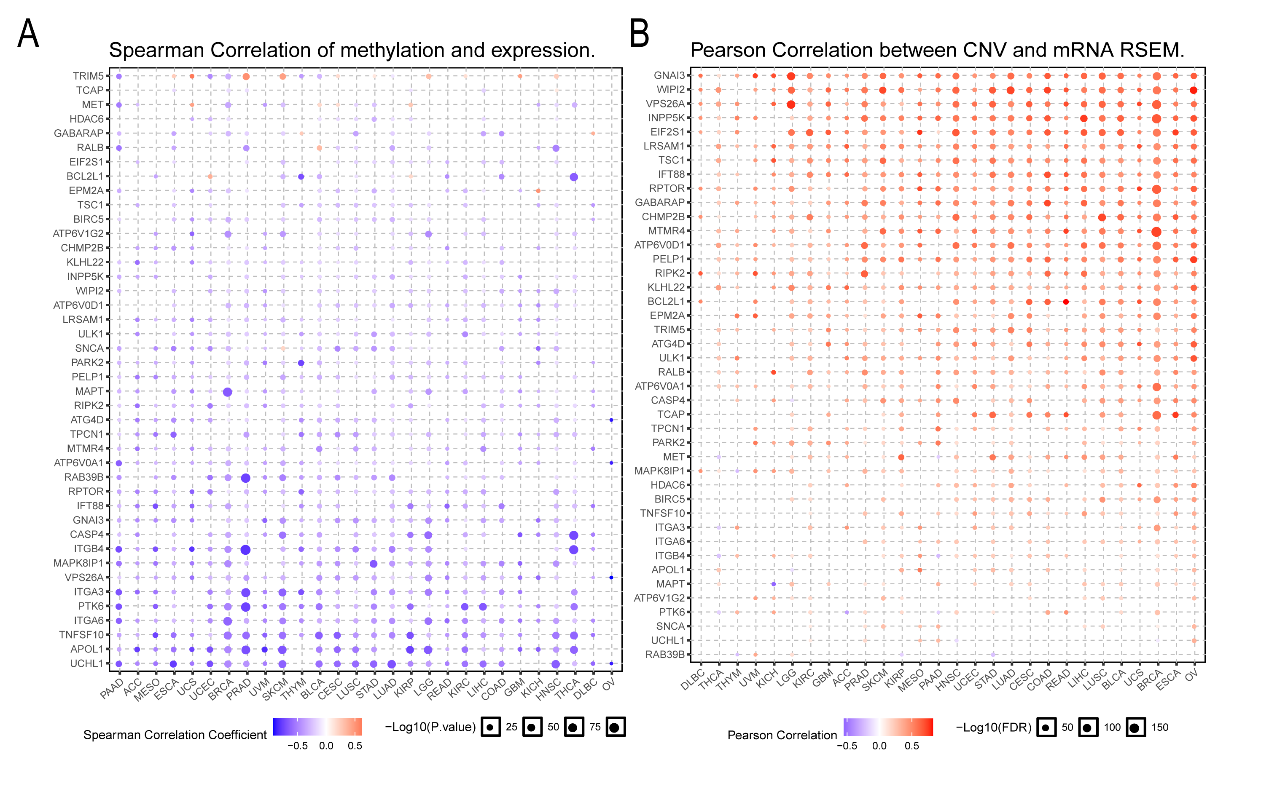


Figure s4


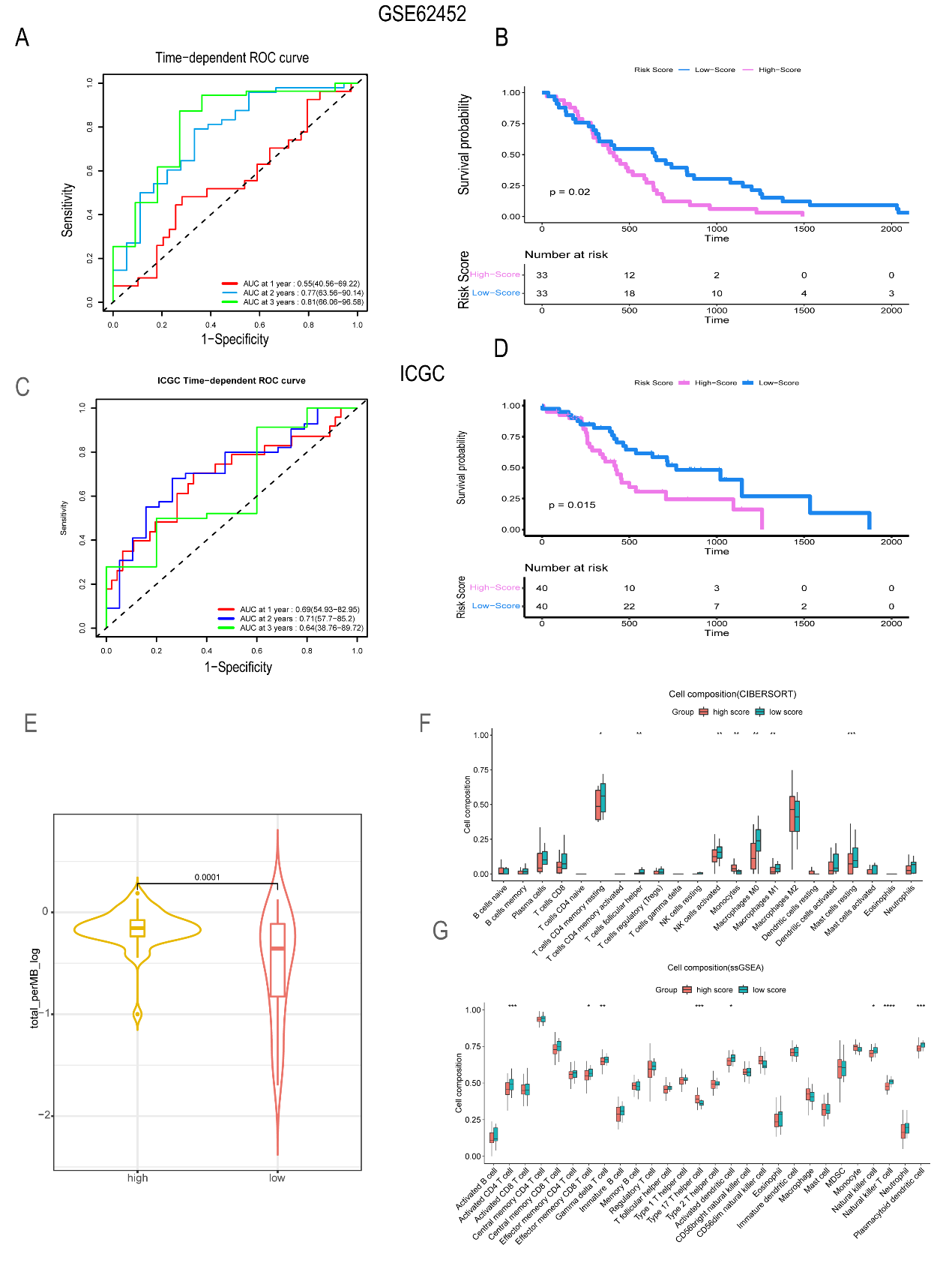


Figure s5


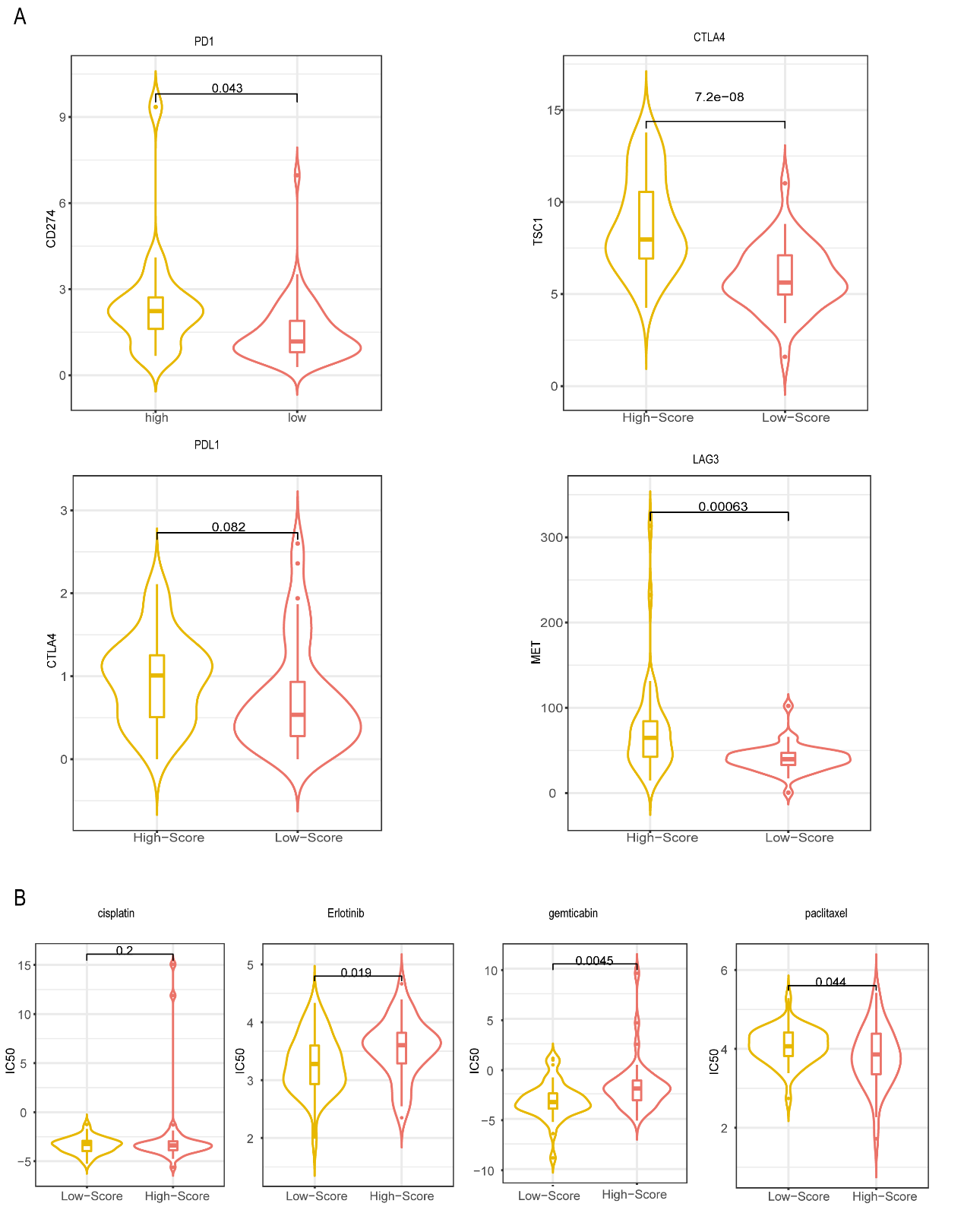


Figure s6


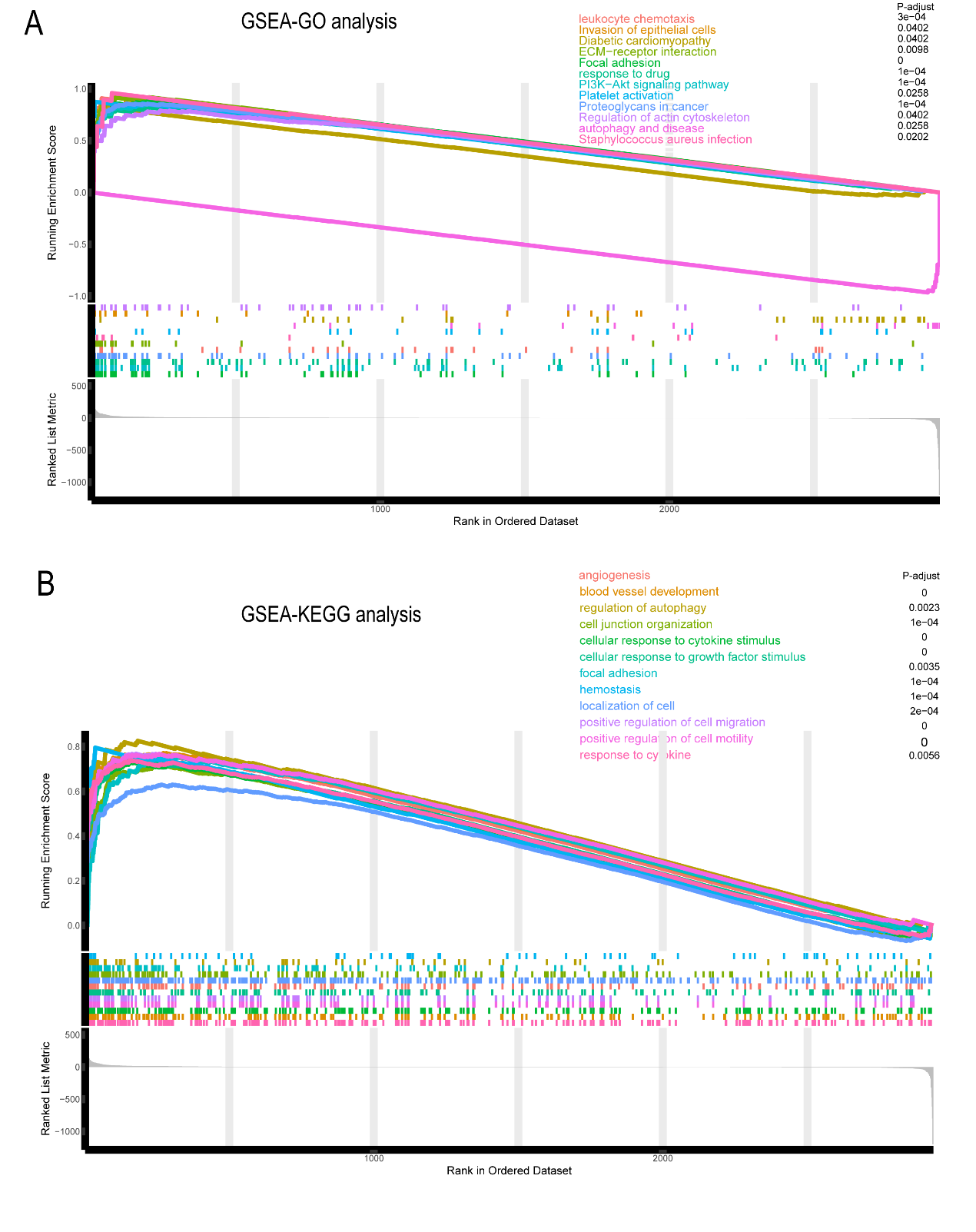


Figure s7


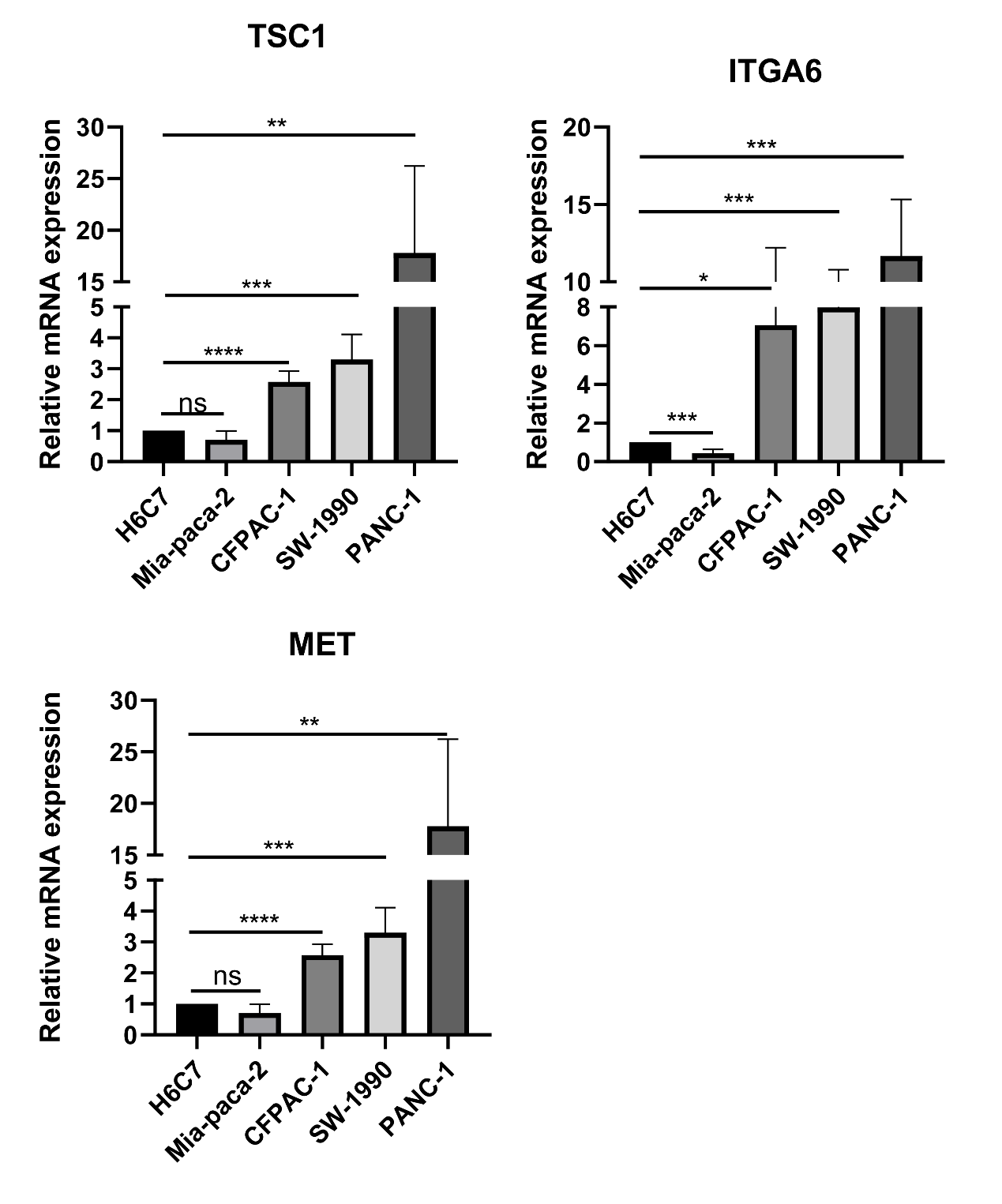


Figure s8


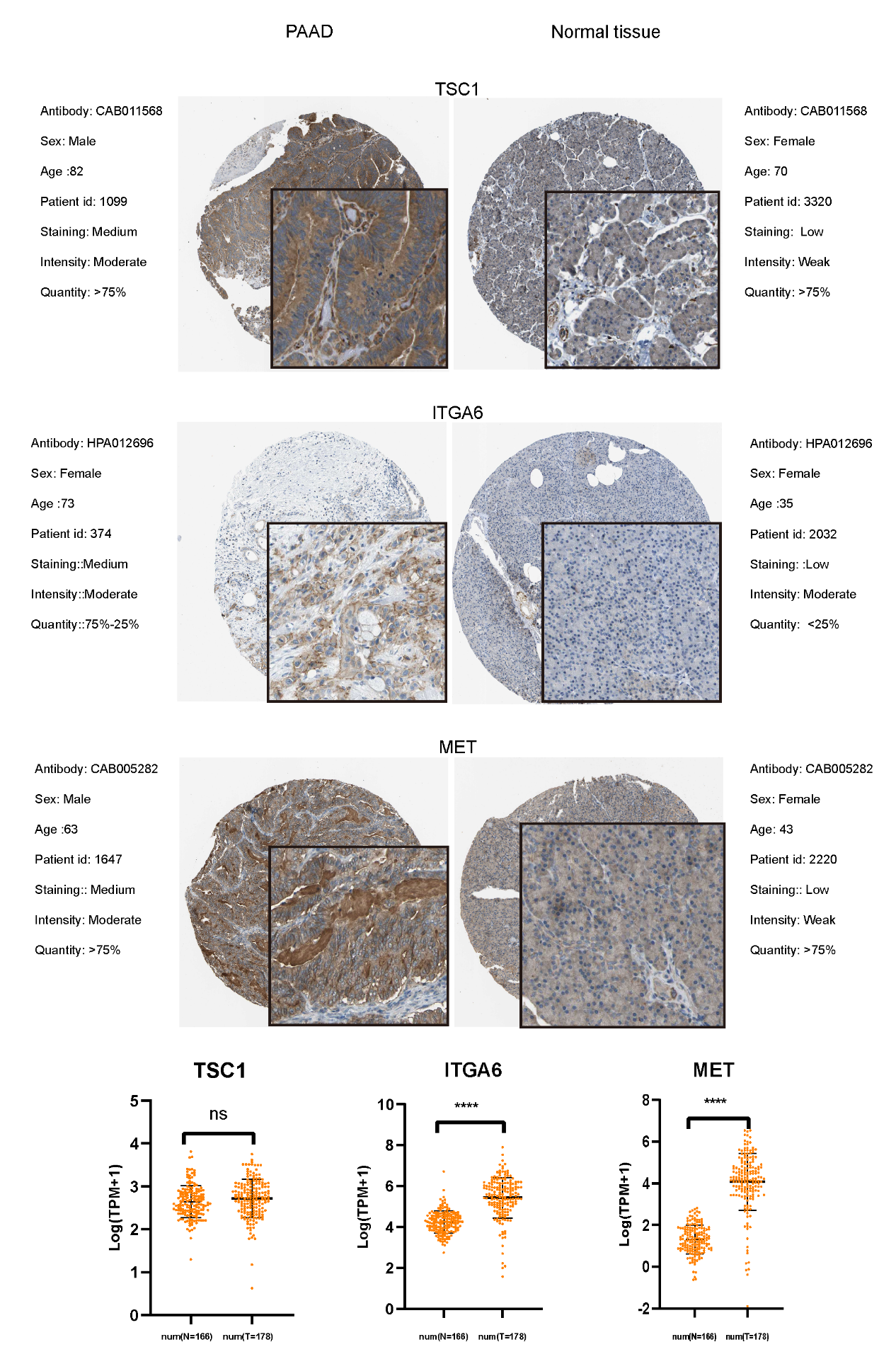

Supplement: Supplementary file 2 — Supplementary Material 2 [file 12920_2022_1371_MOESM2_ESM.docx]
